# (*E*)-2-[2-(4-Chloro­benzyl­idene)hydrazin-1-yl]-4-{[3-(dimethyl­aza­nium­yl)prop­yl]amino}­quinazolin-1-ium bis­(perchlorate)

**DOI:** 10.1107/S1600536812018272

**Published:** 2012-04-28

**Authors:** Nan Jiang, Jian Zuo, Haiyan Wang, Ming Han, Xin Zhai

**Affiliations:** a Key Laboratory of Original New Drug Design and Discovery of the Ministry of Education, Shenyang Pharmaceutical University, Shenyang, Liaoning 110016, People’s Republic of China; b Key Laboratory of Marine Chemistry Theory and Technology, Ministry of Education, College of Chemistry and Chemical Engineering, Ocean University of China, Qingdao, Shandong 266100, People’s Republic of China

## Abstract

In the title compound, C_20_H_25_ClN_6_^2+^·2ClO_4_^−^, the organic cation is roughly planar, as shown by the dihedral angle of 3.78 (3)° between the quinazoline and chloro­phenyl rings. The quinazoline ring is itself approximately planar, with an average deviation of 0.018 (4) Å. The organic cation adopts an *E* configuration with respect to the C= N double bond of the hyrazinyl group. The (dimethyl­aza­nium­yl)propyl­amino side chain is disordered over two sets of sites with occupancies of 0.768 (10) and 0.232 (10). In the crystal, two cations and four anions are linked by strong N—H⋯O hydrogen bonds. Weak C—H⋯O hydrogen bonds exist among these aggregates.

## Related literature

For anti­tumor background to the title compound, see: Abouzid & Shouman (2008 ▶); Zhang *et al.* (2008 ▶); An *et al.* (2010 ▶); Horiuchi *et al.* (2009) ▶. For the structures of closely related compounds, see: Fun *et al.* (2010 ▶); Ferreira *et al.* (2009 ▶); de Souza *et al.* (2010 ▶); Loh *et al.* (2011 ▶).
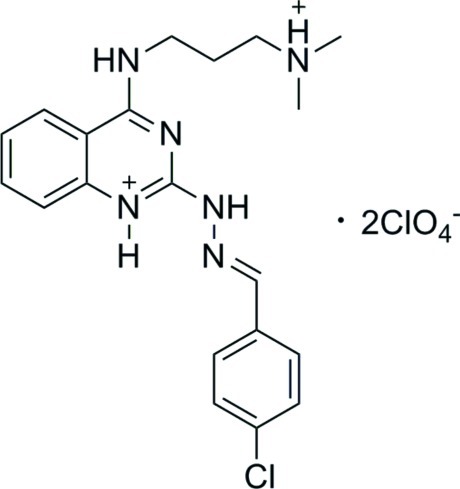


## Experimental

### 

#### Crystal data

C_20_H_25_ClN_6_^2+^·2ClO_4_^−^
*M* *_r_* = 583.81
Triclinic, 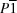

*a* = 10.4533 (18) Å
*b* = 10.5018 (18) Å
*c* = 12.626 (2) Å
α = 104.745 (9)°
β = 91.146 (10)°
γ = 96.21 (1)°
*V* = 1330.9 (4) Å^3^
*Z* = 2
Mo *K*α radiation
μ = 0.40 mm^−1^
*T* = 293 K
0.25 × 0.23 × 0.18 mm

#### Data collection

Siemens SMART CCD area-detector diffractometer
Absorption correction: multi-scan (*SADABS*; Sheldrick, 1996 ▶) *T*_min_ = 0.907, *T*_max_ = 0.932
12435 measured reflections
4889 independent reflections
3979 reflections with *I* > 2σ(*I*)
*R*_int_ = 0.023

#### Refinement

*R*[*F* ^2^ > 2σ(*F* ^2^)] = 0.071
*wR*(*F* ^2^) = 0.219
*S* = 1.09
4889 reflections
371 parameters
62 restraints
H-atom parameters constrained
Δρ_max_ = 0.69 e Å^−3^
Δρ_min_ = −0.92 e Å^−3^

### 

Data collection: *SMART* (Siemens, 1996 ▶); cell refinement: *SAINT* (Siemens, 1996 ▶); data reduction: *SAINT*; program(s) used to solve structure: *SHELXS97* (Sheldrick, 2008 ▶); program(s) used to refine structure: *SHELXL97* (Sheldrick, 2008 ▶); molecular graphics: *SHELXTL* (Sheldrick, 2008 ▶); software used to prepare material for publication: *SHELXTL*.

## Supplementary Material

Crystal structure: contains datablock(s) I, global. DOI: 10.1107/S1600536812018272/im2363sup1.cif

Structure factors: contains datablock(s) I. DOI: 10.1107/S1600536812018272/im2363Isup2.hkl

Supplementary material file. DOI: 10.1107/S1600536812018272/im2363Isup3.cml

Additional supplementary materials:  crystallographic information; 3D view; checkCIF report

## Figures and Tables

**Table 1:** Hydrogen-bond geometry (Å, °)

*D*—H⋯*A*	*D*—H	H⋯*A*	*D*⋯*A*	*D*—H⋯*A*
N1—H1*A*⋯O3	0.91	2.19	2.990 (5)	147
N5—H5*C*⋯O2	0.86	2.11	2.922 (4)	157
N4—H4*C*⋯O1^i^	0.86	2.14	2.964 (4)	160
N2—H2*D*⋯O7	0.86	2.10	2.873 (5)	149
C19—H19⋯O2^ii^	0.93	2.48	3.316 (5)	150
C1—H1*D*⋯O6^iii^	0.96	2.56	3.445 (8)	153
C1—H1*C*⋯O6^iv^	0.96	2.62	3.555 (10)	166

Symmetry codes: (i) 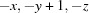; (ii) 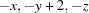; (iii) 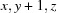; (iv) 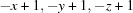.

**Fig. 1.**
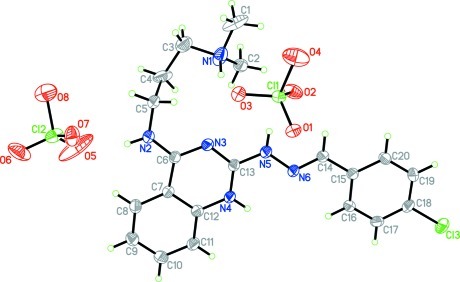
Molecular structure of the title compound, showing 30% probability displacement ellipsoids and the atom-numbering scheme.

**Fig. 2.**
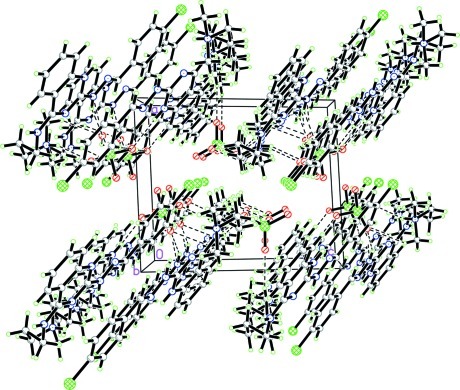
Packing diagram of the title compound.

## supplementary crystallographic information

### Comment

The target compound was designed and synthesized as part of on-going studies aimed at developing antitumor agents based on 4-aminoquinazoline and 4-aminoquinoline nuclei. These have aroused increasing attentions for excellent antitumor potency in recent years, such as gefitinib, the traditional immunostimulatory agents CQ and their derivatives (Abouzid *et al.*, 2008; Zhang *et al.*, 2008; An *et al.*, 2010). With the aim to improve the electron affinity and better biological interactions, a hydrazone fragment was introduced (Horiuchi *et al.*, 2009).

The crystal structure of the title compound is given in Fig. 1. The quinazoline ring is approximately planar, with an average deviation of 0.018 (4) Å. The dihedral angle between the quinazoline ring and the chlorophenyl ring is 3.78 (3) °. The (dimethylazaniumyl)propylamino side chain of the compound is disordered over two sites with occupancies of 0.768 (10) and 0.232 (10), respectively. The cationic part of the compound establishes strong N–H···O hydrogen bonds (N5—H5C–O2, N2—H2D–O7, N1—H1A–O3, N4—H4C–O1, Table 1) with the perchlorate anions. The resulting aggregates of two cations and four anions are linked by weak C–H···O hydrogen bonds (C1—H1C–O6, C1—H1D–O6, C19—H19–O2, Table 1) in the crystal structure (Fig. 2).

### Experimental

Using 2-aminobenzoic acid and urea as the starting materials, (*E*)-*N*-(2-(2-(4-chlorobenzylidene)hydrazinyl) quinazolin-4-yl)-*N'*,*N'*-dimethylpropane-1,3-diamine was prepared according to literature methods (Abouzid *et al.*, 2008; Horiuchi *et al.*, 2009). The compound was purified by silica gel column chromatography (CH_2_Cl_2_/Methanol 15:1). 70% Perchloric acid (24 mmol, 1.96 ml) was added to a solution of the compound (20 mmol, 7.7 g) in acetone (50 ml) at room temperature. Then the reaction mixture was stirred at 313 K for 3 h. After cooling to ambient temperature, the resulting precipitate was filtered and washed with acetone. The resulting solids were dissolved in methanol for 15 days to yield the title compound as colorless single crystals (70% yield).

### Refinement

All H-atoms were positioned geometrically and refined using a riding model, with C—H = 0.96 Å (methyl), C—H = 0.97 Å (methylene), 0.93 Å (aromatic), N—H = 0.86 Å (amine and aromatic), and *U*_iso_(H) =1.2*U*_eq_(C,*N*).

### Crystal data

**Table:**

C_20_H_25_ClN_6_^2+^·2ClO_4_^−^	*Z* = 2
*M**_r_* = 583.81	*F*(000) = 604
Triclinic, *P*1	*D*_x_ = 1.457 Mg m^−^^3^
Hall symbol: -P 1	Mo *K*α radiation, λ = 0.71073 Å
*a* = 10.4533 (18) Å	Cell parameters from 4829 reflections
*b* = 10.5018 (18) Å	θ = 2.5–30.1°
*c* = 12.626 (2) Å	µ = 0.40 mm^−^^1^
α = 104.745 (9)°	*T* = 293 K
β = 91.146 (10)°	Block, colorless
γ = 96.21 (1)°	0.25 × 0.23 × 0.18 mm
*V* = 1330.9 (4) Å^3^	

### Data collection

**Table:**

Siemens SMART CCD area-detector diffractometer	4889 independent reflections
Radiation source: fine-focus sealed tube	3979 reflections with *I* > 2σ(*I*)
Graphite monochromator	*R*_int_ = 0.023
phi and ω scans	θ_max_ = 25.5°, θ_min_ = 1.7°
Absorption correction: multi-scan (*SADABS*; Sheldrick, 1996)	*h* = −12→12
*T*_min_ = 0.907, *T*_max_ = 0.932	*k* = −12→12
12435 measured reflections	*l* = −14→15

### Refinement

**Table:**

Refinement on *F*^2^	Primary atom site location: structure-invariant direct methods
Least-squares matrix: full	Secondary atom site location: difference Fourier map
*R*[*F*^2^ > 2σ(*F*^2^)] = 0.071	Hydrogen site location: inferred from neighbouring sites
*wR*(*F*^2^) = 0.219	H-atom parameters constrained
*S* = 1.09	*w* = 1/[σ^2^(*F*_o_^2^) + (0.1339*P*)^2^ + 1.0689*P*] where *P* = (*F*_o_^2^ + 2*F*_c_^2^)/3
4889 reflections	(Δ/σ)_max_ < 0.001
371 parameters	Δρ_max_ = 0.69 e Å^−^^3^
62 restraints	Δρ_min_ = −0.92 e Å^−^^3^

### Special details

**Table:**

Geometry. All e.s.d.'s (except the e.s.d. in the dihedral angle between two l.s. planes) are estimated using the full covariance matrix. The cell e.s.d.'s are taken into account individually in the estimation of e.s.d.'s in distances, angles and torsion angles; correlations between e.s.d.'s in cell parameters are only used when they are defined by crystal symmetry. An approximate (isotropic) treatment of cell e.s.d.'s is used for estimating e.s.d.'s involving l.s. planes.
Refinement. Refinement of *F*^2^ against ALL reflections. The weighted *R*-factor *wR* and goodness of fit *S* are based on *F*^2^, conventional *R*-factors *R* are based on *F*, with *F* set to zero for negative *F*^2^. The threshold expression of *F*^2^ > σ(*F*^2^) is used only for calculating *R*-factors(gt) *etc*. and is not relevant to the choice of reflections for refinement. *R*-factors based on *F*^2^ are statistically about twice as large as those based on *F*, and *R*- factors based on ALL data will be even larger.

### Fractional atomic coordinates and isotropic or equivalent isotropic displacement parameters (Å^2^)

**Table:**

	*x*	*y*	*z*	*U*_iso_*/*U*_eq_	Occ. (<1)
Cl1	0.32941 (8)	0.74253 (8)	0.10002 (7)	0.0433 (3)	
Cl2	0.26374 (10)	0.25136 (12)	0.61278 (8)	0.0638 (4)	
Cl3	−0.49702 (12)	0.78162 (13)	−0.25924 (10)	0.0702 (4)	
N2	0.2182 (3)	0.4226 (3)	0.3840 (3)	0.0455 (7)	
H2D	0.2213	0.3602	0.4166	0.055*	
N3	0.1121 (3)	0.5197 (3)	0.2718 (2)	0.0391 (6)	
N4	−0.0771 (3)	0.4117 (3)	0.1697 (2)	0.0380 (6)	
H4C	−0.1379	0.4135	0.1234	0.046*	
N5	0.0158 (3)	0.6172 (3)	0.1555 (2)	0.0399 (6)	
H5C	0.0781	0.6806	0.1710	0.048*	
N6	−0.0855 (3)	0.6209 (3)	0.0851 (2)	0.0369 (6)	
O1	0.3001 (3)	0.6543 (3)	−0.0088 (2)	0.0553 (7)	
O2	0.2245 (3)	0.8192 (3)	0.1395 (3)	0.0617 (8)	
O3	0.3692 (3)	0.6777 (3)	0.1811 (2)	0.0582 (7)	
O4	0.4611 (4)	0.8581 (4)	0.0865 (4)	0.1015 (14)	
O5	0.1324 (4)	0.2708 (10)	0.6082 (4)	0.201 (4)	
O6	0.2930 (6)	0.1273 (4)	0.6352 (4)	0.123 (2)	
O7	0.3248 (4)	0.2711 (4)	0.5170 (3)	0.0731 (9)	
O8	0.3333 (5)	0.3762 (5)	0.7247 (4)	0.1072 (14)	
C1	0.3790 (6)	0.9314 (8)	0.3904 (6)	0.094 (3)	0.768 (10)
H1B	0.3551	0.9663	0.3306	0.141*	0.768 (10)
H1C	0.4612	0.8987	0.3783	0.141*	0.768 (10)
H1D	0.3842	1.0001	0.4576	0.141*	0.768 (10)
C2	0.1534 (15)	0.8623 (13)	0.3983 (9)	0.054 (3)	0.768 (10)
H2A	0.0894	0.7915	0.4033	0.081*	0.768 (10)
H2B	0.1373	0.8877	0.3318	0.081*	0.768 (10)
H2C	0.1493	0.9370	0.4601	0.081*	0.768 (10)
N1	0.2784 (4)	0.8189 (4)	0.3980 (3)	0.0711 (11)	0.768 (10)
H1A	0.2818	0.7500	0.3379	0.085*	0.768 (10)
C3	0.3380 (7)	0.7866 (6)	0.4925 (5)	0.071 (2)	0.768 (10)
H3A	0.3252	0.8526	0.5596	0.085*	0.768 (10)
H3B	0.4297	0.7839	0.4841	0.085*	0.768 (10)
C4	0.2729 (9)	0.6522 (6)	0.4957 (5)	0.079 (3)	0.768 (10)
H4A	0.1807	0.6504	0.4836	0.095*	0.768 (10)
H4B	0.2881	0.6397	0.5683	0.095*	0.768 (10)
C5	0.3222 (4)	0.5303 (4)	0.4057 (4)	0.0635 (12)	0.768 (10)
H5A	0.3423	0.5563	0.3390	0.076*	0.768 (10)
H5B	0.3990	0.5033	0.4340	0.076*	0.768 (10)
C1'	0.4014 (16)	0.885 (3)	0.438 (2)	0.081 (7)	0.232 (10)
H1'B	0.4612	0.8206	0.4339	0.121*	0.232 (10)
H1'C	0.3972	0.9346	0.5124	0.121*	0.232 (10)
H1'D	0.4297	0.9437	0.3936	0.121*	0.232 (10)
C2'	0.156 (4)	0.896 (4)	0.397 (4)	0.059 (11)	0.232 (10)
H2'A	0.1676	0.9518	0.3478	0.089*	0.232 (10)
H2'B	0.1452	0.9492	0.4697	0.089*	0.232 (10)
H2'C	0.0805	0.8331	0.3734	0.089*	0.232 (10)
N1'	0.2784 (4)	0.8189 (4)	0.3980 (3)	0.0711 (11)	0.232 (10)
H1'A	0.2844	0.7628	0.3307	0.085*	0.232 (10)
C3'	0.2400 (10)	0.7379 (10)	0.4811 (8)	0.027 (4)	0.232 (10)
H3'A	0.2259	0.7983	0.5508	0.032*	0.232 (10)
H3'B	0.1594	0.6825	0.4552	0.032*	0.232 (10)
C4'	0.3425 (12)	0.6497 (11)	0.4986 (10)	0.033 (4)	0.232 (10)
H4'A	0.4283	0.6954	0.4992	0.040*	0.232 (10)
H4'B	0.3320	0.6261	0.5676	0.040*	0.232 (10)
C5'	0.3222 (4)	0.5303 (4)	0.4057 (4)	0.0635 (12)	0.232 (10)
H5'A	0.3229	0.5634	0.3407	0.076*	0.232 (10)
H5'B	0.4003	0.4881	0.4056	0.076*	0.232 (10)
C6	0.1190 (3)	0.4168 (3)	0.3162 (3)	0.0377 (7)	
C7	0.0215 (3)	0.3013 (3)	0.2901 (3)	0.0377 (7)	
C8	0.0235 (4)	0.1903 (4)	0.3319 (3)	0.0476 (8)	
H8	0.0881	0.1891	0.3833	0.057*	
C9	−0.0688 (4)	0.0830 (4)	0.2980 (3)	0.0523 (9)	
H9	−0.0663	0.0093	0.3258	0.063*	
C10	−0.1655 (4)	0.0850 (4)	0.2224 (3)	0.0535 (9)	
H10	−0.2279	0.0121	0.1998	0.064*	
C11	−0.1714 (4)	0.1935 (4)	0.1798 (3)	0.0491 (9)	
H11	−0.2375	0.1940	0.1297	0.059*	
C12	−0.0763 (3)	0.3026 (3)	0.2132 (3)	0.0384 (7)	
C13	0.0161 (3)	0.5139 (3)	0.1994 (3)	0.0353 (7)	
C14	−0.0831 (3)	0.7243 (3)	0.0498 (3)	0.0398 (7)	
H14	−0.0154	0.7918	0.0722	0.048*	
C15	−0.1847 (3)	0.7380 (3)	−0.0249 (3)	0.0376 (7)	
C16	−0.2904 (3)	0.6397 (3)	−0.0569 (3)	0.0425 (8)	
H16	−0.2963	0.5654	−0.0292	0.051*	
C17	−0.3850 (4)	0.6532 (4)	−0.1289 (3)	0.0478 (9)	
H17	−0.4547	0.5880	−0.1506	0.057*	
C18	−0.3756 (3)	0.7649 (4)	−0.1690 (3)	0.0440 (8)	
C19	−0.2739 (4)	0.8635 (4)	−0.1393 (3)	0.0464 (8)	
H19	−0.2689	0.9374	−0.1676	0.056*	
C20	−0.1798 (3)	0.8500 (3)	−0.0664 (3)	0.0437 (8)	
H20	−0.1113	0.9168	−0.0442	0.052*	

### Atomic displacement parameters (Å^2^)

**Table:**

	*U*^11^	*U*^22^	*U*^33^	*U*^12^	*U*^13^	*U*^23^
Cl1	0.0423 (5)	0.0379 (5)	0.0468 (5)	−0.0048 (3)	−0.0039 (3)	0.0099 (4)
Cl2	0.0543 (6)	0.0819 (8)	0.0474 (6)	−0.0141 (5)	−0.0011 (4)	0.0116 (5)
Cl3	0.0688 (7)	0.0759 (8)	0.0666 (7)	0.0096 (6)	−0.0302 (5)	0.0216 (6)
N2	0.0436 (16)	0.0429 (16)	0.0513 (17)	0.0030 (12)	−0.0128 (13)	0.0165 (13)
N3	0.0363 (14)	0.0390 (15)	0.0406 (15)	0.0044 (11)	−0.0049 (11)	0.0086 (12)
N4	0.0353 (14)	0.0420 (15)	0.0369 (14)	0.0026 (11)	−0.0058 (11)	0.0119 (12)
N5	0.0338 (14)	0.0432 (16)	0.0436 (15)	0.0020 (11)	−0.0071 (11)	0.0144 (12)
N6	0.0329 (13)	0.0410 (15)	0.0375 (14)	0.0041 (11)	−0.0051 (11)	0.0119 (12)
O1	0.0659 (17)	0.0486 (15)	0.0455 (14)	−0.0049 (13)	−0.0153 (12)	0.0072 (12)
O2	0.0433 (15)	0.0480 (16)	0.090 (2)	−0.0004 (12)	0.0120 (14)	0.0124 (15)
O3	0.0685 (18)	0.0532 (16)	0.0528 (16)	−0.0003 (13)	−0.0138 (13)	0.0178 (13)
O4	0.081 (3)	0.082 (3)	0.126 (3)	−0.016 (2)	0.024 (2)	0.008 (2)
O5	0.042 (2)	0.408 (12)	0.098 (4)	0.007 (4)	−0.017 (2)	−0.026 (5)
O6	0.222 (6)	0.058 (2)	0.084 (3)	−0.029 (3)	0.009 (3)	0.029 (2)
O7	0.092 (2)	0.086 (2)	0.0534 (17)	0.0278 (19)	0.0155 (16)	0.0313 (16)
O8	0.091 (3)	0.111 (3)	0.102 (3)	0.006 (2)	−0.008 (2)	−0.001 (3)
C1	0.047 (3)	0.133 (7)	0.067 (4)	0.006 (4)	−0.015 (3)	−0.034 (4)
C2	0.064 (5)	0.042 (8)	0.051 (4)	0.011 (5)	−0.003 (3)	0.002 (4)
N1	0.096 (3)	0.074 (2)	0.0416 (18)	0.044 (2)	−0.0082 (17)	−0.0013 (16)
C3	0.078 (5)	0.075 (4)	0.054 (3)	0.008 (4)	−0.011 (3)	0.006 (3)
C4	0.095 (6)	0.089 (5)	0.037 (3)	−0.045 (5)	−0.012 (3)	0.011 (3)
C5	0.064 (3)	0.056 (2)	0.072 (3)	−0.012 (2)	−0.034 (2)	0.031 (2)
C1'	0.094 (11)	0.083 (10)	0.079 (10)	0.007 (8)	−0.001 (8)	0.048 (9)
C2'	0.037 (11)	0.009 (12)	0.12 (2)	0.001 (8)	0.015 (11)	0.005 (10)
N1'	0.096 (3)	0.074 (2)	0.0416 (18)	0.044 (2)	−0.0082 (17)	−0.0013 (16)
C3'	0.026 (7)	0.016 (6)	0.029 (6)	−0.008 (5)	−0.003 (5)	−0.009 (5)
C4'	0.016 (6)	0.052 (7)	0.028 (6)	−0.018 (5)	−0.016 (5)	0.013 (5)
C5'	0.064 (3)	0.056 (2)	0.072 (3)	−0.012 (2)	−0.034 (2)	0.031 (2)
C6	0.0398 (17)	0.0392 (17)	0.0345 (16)	0.0077 (13)	−0.0010 (13)	0.0095 (13)
C7	0.0377 (17)	0.0405 (18)	0.0348 (16)	0.0041 (13)	−0.0003 (13)	0.0100 (13)
C8	0.050 (2)	0.049 (2)	0.049 (2)	0.0068 (16)	−0.0018 (16)	0.0198 (16)
C9	0.055 (2)	0.048 (2)	0.057 (2)	0.0001 (17)	0.0010 (18)	0.0220 (18)
C10	0.054 (2)	0.046 (2)	0.059 (2)	−0.0074 (17)	−0.0009 (18)	0.0162 (18)
C11	0.0455 (19)	0.054 (2)	0.047 (2)	−0.0047 (16)	−0.0081 (15)	0.0169 (17)
C12	0.0374 (16)	0.0400 (18)	0.0375 (17)	0.0020 (13)	0.0027 (13)	0.0102 (14)
C13	0.0353 (16)	0.0379 (17)	0.0333 (15)	0.0085 (13)	0.0006 (12)	0.0088 (13)
C14	0.0372 (17)	0.0385 (18)	0.0428 (18)	0.0018 (13)	−0.0055 (13)	0.0102 (14)
C15	0.0394 (17)	0.0347 (17)	0.0379 (16)	0.0065 (13)	−0.0014 (13)	0.0073 (13)
C16	0.0475 (19)	0.0337 (17)	0.0449 (18)	0.0031 (14)	−0.0054 (15)	0.0091 (14)
C17	0.049 (2)	0.0392 (19)	0.049 (2)	0.0010 (15)	−0.0108 (16)	0.0037 (15)
C18	0.0437 (18)	0.051 (2)	0.0357 (17)	0.0128 (15)	−0.0069 (14)	0.0059 (15)
C19	0.052 (2)	0.0432 (19)	0.050 (2)	0.0102 (16)	−0.0016 (16)	0.0206 (16)
C20	0.0400 (18)	0.0367 (18)	0.056 (2)	0.0017 (14)	−0.0035 (15)	0.0155 (15)

### Geometric parameters (Å, º)

**Table:**

Cl1—O3	1.444 (3)	C5—H5A	0.9700
Cl1—O2	1.452 (3)	C5—H5B	0.9700
Cl1—O1	1.454 (3)	C1'—H1'B	0.9600
Cl1—O4	1.775 (4)	C1'—H1'C	0.9600
Cl2—O5	1.412 (5)	C1'—H1'D	0.9600
Cl2—O7	1.430 (3)	C2'—H2'A	0.9600
Cl2—O6	1.464 (5)	C2'—H2'B	0.9600
Cl2—O8	1.747 (5)	C2'—H2'C	0.9600
Cl3—C18	1.741 (4)	C3'—C4'	1.538 (10)
N2—C6	1.318 (4)	C3'—H3'A	0.9700
N2—C5	1.450 (5)	C3'—H3'B	0.9700
N2—H2D	0.8600	C4'—H4'A	0.9700
N3—C13	1.330 (4)	C4'—H4'B	0.9700
N3—C6	1.346 (4)	C6—C7	1.461 (5)
N4—C13	1.341 (4)	C7—C12	1.398 (5)
N4—C12	1.392 (4)	C7—C8	1.400 (5)
N4—H4C	0.8600	C8—C9	1.374 (5)
N5—C13	1.338 (4)	C8—H8	0.9300
N5—N6	1.379 (4)	C9—C10	1.382 (6)
N5—H5C	0.8600	C9—H9	0.9300
N6—C14	1.272 (4)	C10—C11	1.384 (6)
C1—N1	1.517 (7)	C10—H10	0.9300
C1—H1B	0.9600	C11—C12	1.404 (5)
C1—H1C	0.9600	C11—H11	0.9300
C1—H1D	0.9600	C14—C15	1.450 (5)
C2—N1	1.430 (16)	C14—H14	0.9300
C2—H2A	0.9600	C15—C20	1.400 (5)
C2—H2B	0.9600	C15—C16	1.404 (5)
C2—H2C	0.9600	C16—C17	1.372 (5)
N1—C3	1.465 (6)	C16—H16	0.9300
N1—H1A	0.9100	C17—C18	1.386 (5)
C3—C4	1.510 (7)	C17—H17	0.9300
C3—H3A	0.9700	C18—C19	1.375 (5)
C3—H3B	0.9700	C19—C20	1.378 (5)
C4—C5	1.620 (7)	C19—H19	0.9300
C4—H4A	0.9700	C20—H20	0.9300
C4—H4B	0.9700		
			
O3—Cl1—O2	110.00 (19)	H5A—C5—H5B	108.6
O3—Cl1—O1	114.28 (17)	H1'B—C1'—H1'C	109.5
O2—Cl1—O1	112.44 (19)	H1'B—C1'—H1'D	109.5
O3—Cl1—O4	107.1 (2)	H1'C—C1'—H1'D	109.5
O2—Cl1—O4	106.45 (18)	H2'A—C2'—H2'B	109.5
O1—Cl1—O4	106.02 (19)	H2'A—C2'—H2'C	109.5
O5—Cl2—O7	110.7 (3)	H2'B—C2'—H2'C	109.5
O5—Cl2—O6	116.7 (5)	C4'—C3'—H3'A	109.0
O7—Cl2—O6	110.9 (3)	C4'—C3'—H3'B	109.0
O5—Cl2—O8	105.3 (3)	H3'A—C3'—H3'B	107.8
O7—Cl2—O8	107.5 (2)	C3'—C4'—H4'A	110.6
O6—Cl2—O8	105.0 (3)	C3'—C4'—H4'B	110.6
C6—N2—C5	122.3 (3)	H4'A—C4'—H4'B	108.7
C6—N2—H2D	118.8	N2—C6—N3	117.7 (3)
C5—N2—H2D	118.8	N2—C6—C7	120.5 (3)
C13—N3—C6	119.0 (3)	N3—C6—C7	121.8 (3)
C13—N4—C12	120.5 (3)	C12—C7—C8	119.3 (3)
C13—N4—H4C	119.7	C12—C7—C6	115.8 (3)
C12—N4—H4C	119.7	C8—C7—C6	124.8 (3)
C13—N5—N6	119.2 (3)	C9—C8—C7	120.7 (4)
C13—N5—H5C	120.4	C9—C8—H8	119.6
N6—N5—H5C	120.4	C7—C8—H8	119.6
C14—N6—N5	116.2 (3)	C8—C9—C10	119.7 (4)
N1—C1—H1B	109.5	C8—C9—H9	120.1
N1—C1—H1C	109.5	C10—C9—H9	120.1
H1B—C1—H1C	109.5	C9—C10—C11	121.2 (4)
N1—C1—H1D	109.5	C9—C10—H10	119.4
H1B—C1—H1D	109.5	C11—C10—H10	119.4
H1C—C1—H1D	109.5	C10—C11—C12	119.2 (4)
N1—C2—H2A	109.5	C10—C11—H11	120.4
N1—C2—H2B	109.5	C12—C11—H11	120.4
H2A—C2—H2B	109.5	N4—C12—C7	119.5 (3)
N1—C2—H2C	109.5	N4—C12—C11	120.8 (3)
H2A—C2—H2C	109.5	C7—C12—C11	119.8 (3)
H2B—C2—H2C	109.5	N3—C13—N5	116.5 (3)
C2—N1—C3	123.2 (6)	N3—C13—N4	123.4 (3)
C2—N1—C1	109.2 (6)	N5—C13—N4	120.1 (3)
C3—N1—C1	97.5 (5)	N6—C14—C15	120.7 (3)
C2—N1—H1A	108.6	N6—C14—H14	119.6
C3—N1—H1A	108.6	C15—C14—H14	119.6
C1—N1—H1A	108.6	C20—C15—C16	118.6 (3)
N1—C3—C4	106.3 (5)	C20—C15—C14	120.6 (3)
N1—C3—H3A	110.5	C16—C15—C14	120.8 (3)
C4—C3—H3A	110.5	C17—C16—C15	120.1 (3)
N1—C3—H3B	110.5	C17—C16—H16	119.9
C4—C3—H3B	110.5	C15—C16—H16	119.9
H3A—C3—H3B	108.7	C16—C17—C18	119.4 (3)
C3—C4—C5	113.7 (5)	C16—C17—H17	120.3
C3—C4—H4A	108.8	C18—C17—H17	120.3
C5—C4—H4A	108.8	C19—C18—C17	122.2 (3)
C3—C4—H4B	108.8	C19—C18—Cl3	118.9 (3)
C5—C4—H4B	108.8	C17—C18—Cl3	119.0 (3)
H4A—C4—H4B	107.7	C18—C19—C20	118.2 (3)
N2—C5—C4	106.7 (4)	C18—C19—H19	120.9
N2—C5—H5A	110.4	C20—C19—H19	120.9
C4—C5—H5A	110.4	C19—C20—C15	121.5 (3)
N2—C5—H5B	110.4	C19—C20—H20	119.3
C4—C5—H5B	110.4	C15—C20—H20	119.3

### Hydrogen-bond geometry (Å, º)

**Table:**

*D*—H···*A*	*D*—H	H···*A*	*D*···*A*	*D*—H···*A*
N1—H1*A*···O3	0.91	2.19	2.990 (5)	147
N5—H5*C*···O2	0.86	2.11	2.922 (4)	157
N4—H4*C*···O1^i^	0.86	2.14	2.964 (4)	160
N2—H2*D*···O7	0.86	2.10	2.873 (5)	149
C19—H19···O2^ii^	0.93	2.48	3.316 (5)	150
C1—H1*D*···O6^iii^	0.96	2.56	3.445 (8)	153
C1—H1*C*···O6^iv^	0.96	2.62	3.555 (10)	166

Symmetry codes: (i) −*x*, −*y*+1, −*z*; (ii) −*x*, −*y*+2, −*z*; (iii) *x*, *y*+1, *z*; (iv) −*x*+1, −*y*+1, −*z*+1.
